# Response to the Comment on “Atom‐Modified gDNA Enhances Cleavage Activity of TtAgo Enabling Ultra‐Sensitive Nucleic Acid Testing”

**DOI:** 10.1002/advs.202417344

**Published:** 2025-03-08

**Authors:** Miaomiao Chen, Xinxin Li, Wenwen Wu, Siyue Lu, Zixiang Liu, Xiaolan Guo, Guangcheng Luo

**Affiliations:** ^1^ Department of Clinical Laboratory, Affiliated Hospital of North Sichuan Medical College School of Laboratory Medicine & Translational Medicine Research Center North Sichuan Medical College Nanchong 637000 China; ^2^ Department of Nephrology Affiliated Hospital of North Sichuan Medical College Nanchong 637000 China

Xin‐min Li et al. recently shared their views regarding our previous study on 2′F‐gDNA‐based TtAgo cleavage activity enhancement. We extend our sincere thanks to the authors for their meticulous work in replicating our experimental data and conducting further investigations. Their contributions have not only affirmed the robustness of our results but have also provided fresh insights into the 2′F‐gDNA mediated TtAgo cleavage. We highly value their alternative viewpoints on the mechanism, as such discussions are crucial for driving scientific progress.

Our previous research introduced an innovatively atom‐modification‐based strategy using 2′F‐gDNA to enhance the cleavage activity of TtAgo. This approach represents a significant departure from traditional methods and offers several advantages. By increasing the interaction between gDNA, TtAgo, and target DNA, we were able to significantly enhance the cleavage activity of TtAgo, making the TtAgo‐based technique more practical and applicable in a wider range of settings. In our original research, we proposed that the enhanced activity of TtAgo by 2′F‐gDNA is associated with increased melting temperature (Tm) and binding affinity. However, we explicitly stated that Tm increase alone does not fully account for the enhanced cleavage activity. We are extremely pleased that the Li's comment has put forward an alternative mechanism, suggesting that the 2′F modification at the 3′‐end of gDNA may impact the propagation step. This hypothesis is both fascinating and worthy of in‐depth exploration.

We are currently engaging in a series of experiments to further investigate the mechanism of 2′F‐gDNA mediated Ago activity enhancement. Our recent studies have shown that 2′F‐gDNA not only enhances the TtAgo's activity but also exhibits a similar effect on PfAgo (unpublished data). Further, our preliminary findings support our perspective that including Tm increase, the 2′F modification might influence the conformational changes during the propagation step, as hypothesized by the comment authors. Additionally, we are exploring the potential role of other factors, such as gDNA length and sequence, in modulating Ago activity. These studies will enhance our understanding of the intricate relationship between 2′F‐gDNA and Ago.

Further, we are excited to announce that our recent studies have shown that 2′F‐gDNA could enhance the activity of PfAgo (unpublished data). This discovery indicates that the enhancing effect of 2′F‐gDNA on Ago proteins may have broad applicability and revolutionize the field of molecular diagnostics and gene editing. We believe that this finding further emphasizes the need for in‐depth research into the mechanism of 2′F‐gDNA.

In response to the comment's suggestions, we are actively optimizing our experimental conditions to improve the robustness of the 2′F‐gDNA/Ago cleavage system. We are exploring the use of alternative modified gDNAs and reaction buffers to enhance Ago activity. Additionally, we are collaborating with other research groups to investigate the further potential mechanisms and applications of the 2′F‐gDNA/Ago system. We look forward to further unlocking the potential of the 2′F‐gDNA/Ago system and promoting its applications in nucleic acid detection and gene editing.

In conclusion, we are grateful for Li's contributions to reproducing our previous results and for the opportunity to engage in a constructive discussion on the proposed mechanism. We look forward to the possibility of collaborating with the comment author and further advancing our understanding in this area. We believe that our research, with its innovative strategies and far‐reaching implications, will continue to make significant contributions to the field.

## Conflict of Interest

The authors declare no conflict of interest.

